# Supplementary material for: Patterns of Intron Gain and Loss in Fungi
Source: PLoS Biol. 2004 Nov 30;2(12):e422. doi: 10.1371/journal.pbio.0020422 (PMC532390; doi:10.1371/journal.pbio.0020422)
Supplement: Table S1 — Also available at http://genes.mit.edu/NielsenEtAl/. (4.3 MB ZIP). [file pbio.0020422.st001.zip › NielsenEtAl/html/297.html]

AN0831.1.NCU03678.1.MG09210.1.FG01852.1


```
 CLUSTAL W (1.82) Multiple Sequence Alignments - Introns Inserted


Sequence 1: NCU03678.1	500 aa
Sequence 2: MG09210.1	504 aa
Sequence 3: FG01852.1	522 aa
Sequence 4: AN0831.1	502 aa
Alignment Length: 614 aa
Number Identitical Residues: 252 aa
Alignment Score (without introns) 11500


MG09210.1 	------------------------------------------------------------
NCU03678.1	------------------------------------------------------------
FG01852.1 	------------------------------------------------------------
AN0831.1  	MRGSEASAPGAFWDAPSTLATSIRTNPPTLETDINTPRLLDSQHVASKFARGMPTASWDQ
          	  .:.:::..:  .:.:: :::  :...: .:. .:.   .:.  ::. : . .::: ..

MG09210.1 	------MAPTNS-IRGVVIGFMSAFGIAVFLAILIVLFYCAKFTTKGRIFLDGIRGPGEY
NCU03678.1	------MPDYRPGMNGIIIGLLSSFGSAILIGCFFLIFYFFRCTTSGRIFLDRIGRPGEY
FG01852.1 	---MGLTSNPSSAFTGVVIGLVSSFGSIVLIALVIFIFWVSGCASTGRIILDRLGRPGEY
AN0831.1  	GNGGSISVSGNSTGKGILIGVLSAFGSAAVAVLVLAAFFFFKYTERGRILLDRMGRPGEF
          	... .      .   *::**.:*:**   .   .:  *:    :  ***:** :  ***:

MG09210.1 	DDEQAFAREEEEALETMDDMQRTEYLRAKA1FIEANPPESMDTQISLSQYLAIQEKGVSA
NCU03678.1	DDEQQFLREEAEALETMDDMQRTEYLRAKA1FIAANPPESLQTDISLSQYLAIQEKGVSA
FG01852.1 	DDEQAFARDEAEALEVMDDMTRQEYLRAKA1WVTANPPESMQTDISLSQYLAIQEKGVSA
AN0831.1  	DDEQAFLREEAEALEVMDDMSRSEYLRSKA1FVEANPPESVQTDISLSQFLAIQEKGVSA
          	**** * *:* ****.**** * ****:** :: ******::*:*****:**********

MG09210.1 	WEFEPELEIANCFVEARTEIEFFDSECCVLSNLPVPKQNEVYYWEAKIYEKPETTLMSIG
NCU03678.1	WEFEPELEIANCFVEARTEIEFFDSECTVMSNLPVPKQNEVYYWEAKIYDKPENTLISIG
FG01852.1 	WEFEPELEIANCFVEARTEIEFFDSECTVMSNLPVPKQNDVYYWEAKIYEKPESTLLSIG
AN0831.1  	WEFQPELEIANCFVEARTEIEFYDSICSVQTNLPVPKQNDVYYWEAKIFDKPETTLISIG
          	***:******************:** * * :********:********::***.**:***

MG09210.1 	MSTKPYPLFRLPG1YHKHSIAYLSNGTRRYNQPFKGTNYGPKFVQGDVIGVGYRPRTGAI
NCU03678.1	MATKPYPLFRLPG1FHKYSVAYLSNGTRRYNQPFNATSYGPQVVQGDVIGVGYRPRSGTI
FG01852.1 	MATKPYPLFRLPG1YHKYSVGYQSTGARRYNQPFGATPYGPPLVQGDVVGVGYRPRTGAI
AN0831.1  	MSTKPYPLFRLPG1YHKSSVAYESTGHRRYNQPFTSLPYGPQLTQGDVVGVGYRPRSGTI
          	*:*********** :** *:.* *.* ******* .  *** ..****:*******:*:*

MG09210.1 	FFTRNGKRLEDVAQGLKTQNFFPSIGANGPCIVHVNFGQAGFVFIEANVKKWGLAPMNGS
NCU03678.1	FFTRNGKKLEDVVHGLKSQNFFPSIGANGPCIVHVNFGQAGFVFIEANVKKWGLAPMTGS
FG01852.1 	FFTRNGKKLEEVVHGLKAQNFFPAIGANGPATIHVNLGQAGFVFIEANVKKWGLAPVTGS
AN0831.1  	FFTRNGKKLEDVVHGQKSQNFFPTVGANGPCTVHVNFGQLGFVFIEANVKKWGLAPMTGS
          	*******:**:*.:* *:*****::*****. :***:** ****************:.**

MG09210.1 	LAPPPPYGSEQGSILLESGRKDGYAP--ARGHSQSVSVLPY-SRQ-QHPVDTRHGRSRSG
NCU03678.1	LAPPPPYGSEQGSILLEAGTKDGFTSTLGRGRGYSHPVQTY-SRQGLAAVDSSHNRTRSG
FG01852.1 	LAPPPPYGSEQGSILLEAGTKDGSTYPQGRQHSHSITAGSYNTRNPSNDLVPQHGRTRSG
AN0831.1  	LAPPPPYGSEQGSILLESG----------RESAAHVAHRVY--------QDPSNAQTNS-
          	*****************:*          *  .   .   *          . : ::.* 

MG09210.1 	NFRSLPPTSPGPVRSPTDISLA---HLIPTDEVGEPSRNPTTYSTGTSSSSAVPADQSLT
NCU03678.1	NFRMFPPTSPGPVRSPTDISLA---HLVPTEDAGEPS-----------SSAAALVDQ---
FG01852.1 	NFRVLPPTSPGPVRSSTDISLA---HFVPNEESGEAS------------SNAGPQQE---
AN0831.1  	TVRIPPSVSPGPIRSPTDISLAPLAHIPSHDDAGEGS------------SRTGPSNP---
          	..*  *..****:**.******. :*: . :: ** *            * :   :    

MG09210.1 	RSNLPPLGLGLDSSQHAPPPEYSSP--TTSETSGSRRNSFESDNAPLISITNRSRGASSA
NCU03678.1	--DGQPITGGFLSP-DQPPPEYTSP--VNSRP-GSRRHSSDSENTPLIQLSGRSRGASSA
FG01852.1 	THNHNPLHLHLEDA-TNPPPEYQSPDHSSSDDDDDRYGSDSEEDTPLIRVMNRSRGNSSA
AN0831.1  	--EQVPL-LNPDELEQVPPPEYTSP--------------------------DGSPRASQD
          	  :  *:     . .  ***** **                          . *   *. 

MG09210.1 	TVRSGRSG-------TGSRPPHSPPIPSYNDAVREGAGRDRSDSA--------MSGR---
NCU03678.1	TARPIQSGGSHNRPRAGSHRPPSPPIPSYQDAVRQGAGRDRSDST--------RSARDSS
FG01852.1 	TVLPSD----------RSSSPRNPPVPSYSDAVRQGAGRDRSDSARLPPPNRTASNRPRS
AN0831.1  	FSREGQ-----------------PPIPSYDAAVGNPADNTSHTH----------------
          	                       **:***. ** : *..                     

MG09210.1 	-~---------------
NCU03678.1	-~---------------
FG01852.1 	N1RKPIRPLSRGEIGWI
AN0831.1  	-~---------------
          	
```
